# Expanding Horizons: Increasing Injectable Vaccine in the Expanded Program on Immunization

**DOI:** 10.31729/jnma.8773

**Published:** 2024-11-30

**Authors:** Birendra Prasad Gupta, Namita Ghimire

**Affiliations:** 1 Virology Society Nepal, Kathmandu, Nepal; 2 Nepal Health Research Council, Ramshah Path, Kathmandu, Nepal

**Keywords:** *expanded program on immunization*, *immunization*, *injection*, *vaccine*

## Abstract

The World Health Organization started the Expanded Program on Immunization in 1974, including 6 antigens with up to 8 vaccine doses for childhood vaccination. The number of antigens has now increased to 11 antigens in 21 vaccines. The expansion of vaccination programs to include more antigens and doses has led to concerns about the burden of multiple injections on infants and children, as well as factors such as fear of adverse reactions, pain, and overall acceptability of vaccines. To address these challenges, there's a call for research to focus on developing combined vaccines that can be administered through more acceptable routes, such as oral, nasal, or needleless administration. This approach could potentially reduce the number of injections required and increase the willingness of individuals to receive vaccines. We highlight the importance of ongoing research and innovation in vaccine development and delivery methods to ensure that vaccination programs remain effective, efficient, and acceptable to the communities they serve, particularly in resource-limited settings.

## INTRODUCTION

Immunizations are among the most cost-effective health interventions with estimates of an 18% rate of return by 2020 for investments to widely introduce new and under-utilized vaccines.^1^ The expansion of vaccine development and delivery efforts is vital for reducing mortality and morbidity worldwide, at the same time the present challenges associated with an increasing number of injectable vaccines are a rising concern.^2^

## ANALYSIS OF THE NUMBER OF VACCINE INJECTIONS BEING USED IN LMICS

**Figure 1:**
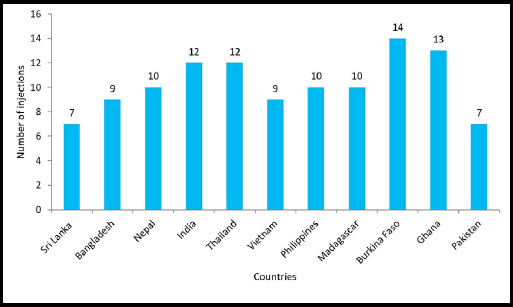
Number of injections by country from birth to 24 months.

We conducted a comparative analysis of the number of vaccine injections used in various countries, within different WHO regions, focusing on low- and middle-income countries (LMICs). The data were acquired from the global summary available on the WHO vaccine-preventable diseases website 2017.^2,3^ The analysis provides valuable insights into the diversity of vaccine schedules and highlights potential areas for improvement or harmonization across regions. By reviewing the introduction of new vaccines into the Expanded Program on Immunization (EPI) schedules of representative countries within each region, we can track the evolution of vaccination programs and assess the burden of multiple injections on children from birth to two years of age. We analyze data from Southeast Asia (Sri Lanka, Bangladesh, Nepal, India, and Thailand), the Western Pacific (Vietnam and Philippines), Africa (Madagascar, Burkina Faso and Ghana) and the Eastern Mediterranean region (Pakistan) (Figure 1), for a comprehensive understanding of vaccination practices across different contexts within each WHO region. This comparative analysis can help identify trends, disparities, and challenges in vaccine delivery and uptake, as well as inform strategies for optimizing immunization programs to ensure maximum coverage and effectiveness.^3-5^

**Figure 2:**
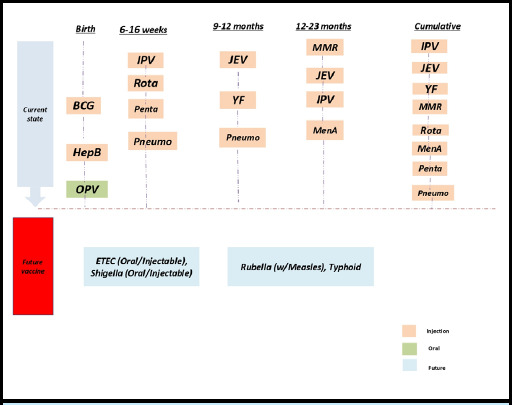
Vaccines recommended in the WHO immunization schedule for infants and toddlers. BCG=Bacillus Calmette-Guerin Vaccine, HepB=Hepatitis B Vaccine, OPV=Oral Polio Vaccine, IPV=Injectable Polio Vaccine, Rota=Rotavirus Vaccine, Penta=Pentavalent Vaccine, Pneumo=Pneumococcal Vaccine, MenA=Meningococcal A vaccine, ETEC=Enterotoxicogenic E.Colli

The introduction of combination vaccines like pentavalent (DTwPHibHepB) and measles-mumps-rubella (MMR) or measles-rubella (MR) has indeed helped to reduce the number of individual injections required for children in LMICs. However, despite these improvements, the total number of needle injections remains high, ranging from 7 to 11 from birth to two years of age depending on the country's adoption of vaccines.^5-7^ Vaccines recommended in the WHO immunization schedule for infants and toddlers include various vaccines (Figure 2).

In Burkina Faso, for instance, children are currently receiving 11 injections according to the recent EPI schedule, with infants receiving 9 injections and toddlers receiving 5 vaccines (Figure 1). Additionally, in countries like India, where an additional MR vaccine is administered at 15-18 months, the total number of injections can be even higher (Figure 1). The increasing number of injections raises concerns among parents about potential pain, adverse events following multiple injections, and perceived stress on the immune system of children.^8^ To address these concerns and overcome the challenges associated with multiple injections, scientists are working to discover and develop new methods of vaccine delivery.

## ADDRESSING CHALLENGES FOR MULTIPLE VACCINE INJECTIONS

Vaccines indeed stand as one of the most successful and cost-effective public health interventions, especially in preventing childhood deaths due to infectious diseases.^9^ The introduction of new injectable vaccines in many LMICs, often as part of global health initiatives, reflects the ongoing commitment to improving public health outcomes worldwide.^10^ However, the increased number of injectable vaccines poses challenges, particularly in LMICs, where more infants are receiving multiple injections during the same immunization visit. This raises concerns about the acceptability and potential effects of this practice on the outcomes of immunization programs in these countries. While some studies have explored the acceptability of multiple vaccine injections in high-income countries, there is limited empirical evidence from LMICs to guide decision-making in this regard. In various countries across different WHO regions, a fully immunized child now requires between nine and 13 antigens and may receive between 7 and 11 injections by the age of 2 (Figure 1). These vaccines protect a wide range of infectious diseases, including measles, mumps, rubella, varicella, hepatitis B, diphtheria, tetanus, pertussis (DTaP), Haemophilus influenzae type b (Hib), polio, influenza (flu), rotavirus, and pneumococcal disease (Figure 2). Despite the availability of combination vaccines, multiple injections are still required at several immunization visits to deliver the recommended antigens. To address this challenge, additional research should focus on generating combined vaccines administered through more acceptable routes, such as oral, nasal, or needleless administration, in the future. By investing in innovative vaccine delivery methods and addressing concerns surrounding multiple injections, we can further enhance the effectiveness and acceptability of immunization programs in LMICs, ultimately contributing to better public health outcomes globally. The major concern is highlighted :

**Acceptance:** Vaccine acceptance is influenced by various factors, including individual beliefs, social influences, and access to information. Some of key elements that affect vaccine acceptance are trust in healthcare providers, strong trust between patients and healthcare providers can significantly enhance acceptance; open and transparent communication about the benefits and risks of vaccines fosters trust. Promoting vaccine acceptance requires a multifaceted approach that considers trust, education, accessibility, and social influences. By addressing these factors, health authorities can work towards improving vaccination rates and overall public health. ^11^

**Vaccine Safety Monitoring:** Vaccine safety monitoring is a dynamic and ongoing process that plays a crucial role in maintaining public trust and ensuring the benefits of vaccination outweigh any risks. By employing a combination of pre- and post-licensure safety measures, health authorities can effectively manage vaccine safety and address public concerns.^12^

**Combination Vaccines:** Combination vaccines, which combine multiple vaccines into a single shot, offer several benefits and considerations like reduced number of injections which reduces the number of shots a child needs, making it more convenient for patients and parents. In addition, Fewer injections may lead to higher vaccination rates, can lower healthcare costs by reducing the number of visits needed and minimizing administrative expenses, simplified Immunization Schedule which help streamline vaccination schedules, making it easier for healthcare providers and families to keep track of immunizations and enhanced Immunogenicity^13,14^ Some combination vaccines are MMR (measles, mumps, and rubella vaccines), DTaP Vaccine (Combines diphtheria, tetanus, and pertussis vaccines), Pentavalent Vaccine (Combines DTaP, Haemophilus influenzae type b, inactivated poliovirus vaccines. The combination vaccine approach is a practical strategy that can enhance immunization efforts. By addressing safety, public perception, and clinical guidelines, health authorities can maximize the benefits of these vaccines while ensuring community health.^15^

**Policy Implementation:** Effective policy implementation for vaccine acceptance requires collaboration, community engagement, and ongoing evaluation. By addressing the various factors influencing vaccine perceptions and making vaccines accessible and trusted, public health officials can significantly enhance vaccination rates and overall community health.^16,17^

**Education,Communication and public engagement:** It includes providing clear and accurate information to parents and caregivers about the importance of vaccination and addressing concerns about the number of injections and potential side effects. Public engagement for vaccines is crucial in promoting vaccine acceptance and uptake which can be obtained using some effective strategies like Education Campaigns to provide clear, accessible information about how vaccines work, their safety, and their benefits; Community Involvement by partner with local organizations, healthcare providers, and community leaders to reach diverse populations which builds trust and encourages dialogue. Effective public engagement requires a multi-faceted approach that respects community values and prioritizes transparency and trust.^18,19^

By addressing these challenges and investing in research and development of new vaccine delivery methods, we can work towards improving the acceptability, safety, and effectiveness of immunization programs in LMICs.

## WAY FORWARD

To successfully integrate additional injectable vaccines into the EPI, a coordinated and well-planned approach is required. This will involve strategic investments in health infrastructure, including the expansion of cold chain capabilities, the provision of adequate resources for healthcare worker training, and the development of targeted communication strategies to address vaccine hesitancy.

Partnerships with international organizations, such as Gavi, the Vaccine Alliance, and the World Health Organization, will be crucial in securing the necessary funding and technical support. Leveraging these partnerships can help Nepal overcome logistical and financial barriers to ensure that the expanded vaccine schedule reaches all corners of the country.

## CONCLUSIONS

The expansion of injectable vaccines in Nepal's EPI offers a significant opportunity to protect the population against a broader range of diseases. While challenges exist, they can be addressed through strategic investments in infrastructure, workforce development, and public engagement. As the health landscape continues to evolve, Nepal's immunization program must remain responsive to emerging threats, positioning itself to safeguard the health of its population and contribute to global disease prevention efforts.

## References

[1] Desalew A, Semahegn A, Birhanu S, Tesfaye G (2020). Incomplete Vaccination And Its Predictors Among Children In Ethiopia: A Systematic Review And Meta-Analysis.. Glob Pediatr Health..

[2] World Health Organization. Global Reported Cases Of Vaccine-Preventable Diseases (Vpds) [Internet].. Immunization dashboard Global.

[3] Kolasa MS, Bisgard KM, Prevots DR, Desai SN, Dibling K (2016). Parental Attitudes Toward Multiple Poliovirus Injections Following A Provider Recommendation.. Public Health Rep..

[4] Duijzer E, van Jaarsveld W, Dekker R (2018). Literature Review-The Vaccine Supply Chain.. European Journal of Operational Research..

[5] Lieu TA, Davis RL, Capra AM, Mell LK, Quesenberry CP (2001). Variation In Clinician Recommendations For Multiple Injections During Adoption Of Inactivated Polio Vaccine.. Pediatrics.

[6] Chapagain RH, Adhikari S, Giri BR, Ray P, Shrestha NJ, Prajapati B (2022). Factors Affecting Willingness To Participate In Vaccine Clinical Trials In An Underdeveloped Country: Perspective From Nepal.. Hum Vaccin Immunother..

[7] Orenstein WA, Cairns L, Hinman A, Nkowane B, Olive J-M, Reingold AL Measles And Rubella Global Strategic Plan 2012-2020 Midterm Review Report.. Vaccine..

[8] Kovacs G, Norman R (2018). How to Improve Preconception Health to Maximize IVF Success [Internet]..

[9] Saluja T, Rai GK, Chaudhary S, Kanodia P, Giri BR, Kim DR (2022). Immune Non-Interference And Safety Study Of Vi-Dt Typhoid Conjugate Vaccine With A Measles, Mumps And Rubella Containing Vaccine In 9-15 Months Old Nepalese Infants.. Vaccine..

[10] Saluja T, Giri BR, Chaudhary S, Tamrakar D, Kanodia P, Palkar S (2021). Challenges And Opportunities In Setting Up A Phase Iii Vaccine Clinical Trial In Resource Limited Settings: Experience From Nepal.. Hum Vaccin Immunother..

[11] Bhattarai M, Baniya JB, Aryal N, Shrestha B, Rauniyar R, Adhikari A (2018). Epidemiological Profile And Risk Factors For Acquiring HBV And/Or HCV In HIV-Infected Population Groups In Nepal.. Biomed Res Int.

[12] Bhutta ZA, Ali S, Cousens S, Ali TM, Haider BA, Rizvi A (2008). Interventions To Address Maternal, Newborn, And Child Survival: What Difference Can Integrated Primary Health Care Strategies Make?. The Lancet..

[13] World Health Organization. (2013). Global tuberculosis report 2013 [Internet]..

[14] Sah SK, Gonzalez JV, Shrestha S, Adhikari A, Manandhar KD (2018). Human Papillomavirus Genotype Distribution In Cervical Cancer Biopsies From Nepalese Women.. Infect Agent Cancer.

[15] Kumar Rai G, Saluja T, Chaudhary S, Tamrakar D, Kanodia P, Giri BR (2022). Safety And Immunogenicity Of The Vi-Dt Typhoid Conjugate Vaccine In Healthy Volunteers In Nepal: An Observer-Blind, Active-Controlled, Randomised, Non-Inferiority, Phase 3 Trial.. Lancet Infect Dis..

[16] Bedford H, Lansley M (2007). More Vaccines For Children? Parents' Views.. Vaccine..

[17] Gupta BP, Tuladhar R, Kurmi R, Manandhar KD (2018). Dengue Periodic Outbreaks And Epidemiological Trends In Nepal.. Ann Clin Microbiol Antimicrob..

[18] Gupta BP, Shrestha A, Adhikari A, Lama TK, Sapkota B (2016). Acute Hepatitis E Virus Infection In Human Immunodeficiency Virus-Positive Men And Women In Nepal: Not Quite A Rare Entity.. Hepatology..

[19] Tamrakar D, Poudel P, Thapa P, Singh S, Khadgi A, Thapa S (2024). Safety And Immunogenicity Of Conjugate Vaccine For Typhoid (Vi-Dt): Finding From An Observer-Blind, Active-Controlled, Randomized, Non-Inferiority, Phase Iii Clinical Trial Among Healthy Volunteers.. Hum Vaccin Immunother..

